# Quality of Life in Lymphedema Patients Treated by Microsurgical Lymphatic Vessel Transplantation—A Long-Term Follow-Up

**DOI:** 10.3390/life14080957

**Published:** 2024-07-30

**Authors:** Louisa Antonie Hock, Tim Nürnberger, Konstantin Christoph Koban, Paul Severin Wiggenhauser, Riccardo Giunta, Wolfram Demmer

**Affiliations:** Department of Hand, Plastic and Aesthetic Surgery, LMU Klinikum, Ziemssenstraße 1, 80336 Munich, Germany

**Keywords:** lymphoedema, microsurgery, lymphatic vessel transplantation, health-related quality of life, HRQoL

## Abstract

Introduction: Lymphedema is a chronic condition characterized by the accumulation of lymph fluid in the upper or lower extremities, leading to swelling, discomfort, and disability in everyday life. While various treatment modalities exist, microsurgical lymphatic vessel transplantation (LVT) has emerged as a promising option. However, there is little to no long-term follow-up data regarding patients’ improvement in quality of life for this surgical technique. The present study conducts an investigation of the long-term health-related quality of life (HRQoL) over more than 20 years in patients with lymphedema treated with LVT and accomplishes this by utilizing an adapted SF-12 survey. Patients and methods: A retrospective analysis was conducted on patients who underwent LVT between 1 January 1983 and 1 October 2010 at LMU Clinic Munich (*n* = 35). Quality of life scores were assessed preoperatively and today in terms of physiological conditions, psychological conditions, and burden of therapy using a SF-12 survey adapted to the symptoms and impairments that chronic lymphedemas are known to cause. Results: Our findings demonstrate a significant improvement in HRQoL following LVT, with notable enhancements in physiological and psychological conditions such as burden of therapy. Physiological conditions showed a significant positive change of 3.2648 (*p* < 0.01). Psychological conditions improved significantly by a factor of 2.0882 (*p* < 0.01). Additionally, the burden of therapy improved significantly by 1.5883 points (*p* < 0.01). Conclusion: Previous studies have already shown a significant improvement of HRQoL within the first postoperative years for patients treated by LVT. This study also demonstrates significant long-term improvement after LVT, thus underlining the effectiveness of using LVT to improve the quality of live for patients with both primary and secondary lymphedema long-term.

## 1. Introduction

The lymphatic system, with lymphatic vessels as conduits, is the second most important transport system in the human body, next to the blood circulation. It specializes in transporting fluid, proteins, antigens, lymphocytes and other cells of the immune system, and chylomicrons, and furthermore, it directs pathogens such as bacteria and foreign bodies to the lymph nodes where they are presented to the immune system [1,2,3,4]. In healthy individuals, the transport capacity of lymphatic vessels exceeds the required lymphatic load of the tissue by a factor of ten [5]. Therefore, the healthy human body is capable of transporting the lymph fluid sufficiently, averting lymphatic accumulation and stasis and thus preventing the development of lymphoedema [2].

Lymphoedema manifests when the transport capacity for lymphatic drainage from tissue is inadequate to meet the demand, which can arise from either primary or secondary causes [6]. Secondary lymphedema is significantly more common than primary lymphedema. The prevalence of secondary lymphedema is around 1/1000, while that of primary lymphedema is around 1/100,000 [7,8]. Secondary lymphedema often results from the removal of local lymph nodes or the destruction of lymphatic vessels [9]. Without adequate therapy, lymphoedema progresses through three stages (I–III) and becomes an irreversible chronic condition [7]. Known physiological manifestations of chronic lymphoedema include the fibrosclerotic remodeling of connective tissue, pathological proliferation of fat and connective tissue with resulting pain, and an increased predisposition to acute skin infections such as erysipelas [10,11,12]. In addition, manifest lymphoedema also leads to biopsychosocial limitations. The quality of life of patients with chronic lymphoedema is significantly lower compared to healthy individuals [13]. Patients are restricted in their physical and social functioning, vitality, and mental well-being [14]. The often massively swollen extremities are both an aesthetic burden for the patients and a significant barrier to everyday activities [15]. The prevalence of psychological comorbidities, such as depression or anxiety disorders, is therefore increased in patients with chronic lymphoedema [16].

It is estimated that lymphoedema affects around 140 to 200 million people worldwide [17]. The Bonn Vein Study 2003 estimated the prevalence of lymphoedema in Germany at approximately 1.5–2.0% [18]. Even though the treatment options for the primary diseases such as mammary carcinoma have progressed, secondary lymphoedema remains a common complication of cancer treatment [19]. Thus, there is an interest in treating and alleviating the high level of suffering caused by this disease.

In the early phase, conservative treatment including decongestive therapy, skin care, and manual lymphatic drainage usually suffice. In cases where patients do not respond to these measures, surgical therapy may become necessary [20]. Currently, different microsurgical therapies are in use [17,21]. The microsurgical transfer of single lymph vessels to bridge localized lymphatic blockade is an established method in lymphatic surgery [22,23,24]. A comparison of permeability for autologous lymphatic vessel transplants (LVT), allogeneic lymphatic vessel transplants, autologous vein transplants, and expanded polytetrafluoroethylene (ePTFE) microprostheses found autologous lymphatic vessel transplants to be the superior surgical treatment option in chronic lymphedema [20]. A significant improvement in health-related quality of life through microsurgical autologous lymphatic vessel transplantation within the first few postoperative years has already been demonstrated [25]. While current literature suggests this microsurgical therapy to be a good option for the treatment of manifest secondary lymphoedema, there is little to no long-term follow-up data regarding patients’ improvement in quality of life for this surgical technique. The usual objective indicators do not always reflect the postoperative success, especially with regard to health-related quality of life (HRQoL) of patients [26]. In recent years, more comprehensive patient-reported outcome measures (PROMs), which combine physical and psychological aspects, are used to assess the results of surgical interventions [27]. The aim of this study is to examine the long-term results of LVTs in patients with lymphoedema in regard to their HRQoL. The results are intended to help surgeons make treatment decisions in the future and improve the management of chronic lymphoedema. As many patients experience major restrictions in their HRQoL, as described above, these aspects should be given greater consideration in future surgical treatments. The intention is to reduce the enormous psychological distress experienced by many patients with lymphoedema.

## 2. Material and Methods

This retrospective study assesses the HRQoL in patients diagnosed with lymphedema of both lower and upper limb who have been treated by microsurgical autologous LVT. Patients diagnosed with lymphedema were recruited from the Department of Hand, Plastic and Aesthetic Surgery of the LMU Clinic Munich.

The inclusion criteria for patients involved having received microsurgical therapy for a manifested lymphoedema through LVT from the Department of Hand, Plastic, and Aesthetic Surgery of the LMU Clinic Munich or its precursor organization the Section of Plastic Surgery, Hand Surgery of the Microsurgical and Surgical Clinic Großhadern between 1 January 1983 and 31 October 2010. All patients aged 18 years and older who agreed to participate were included.

**Figure 1:**
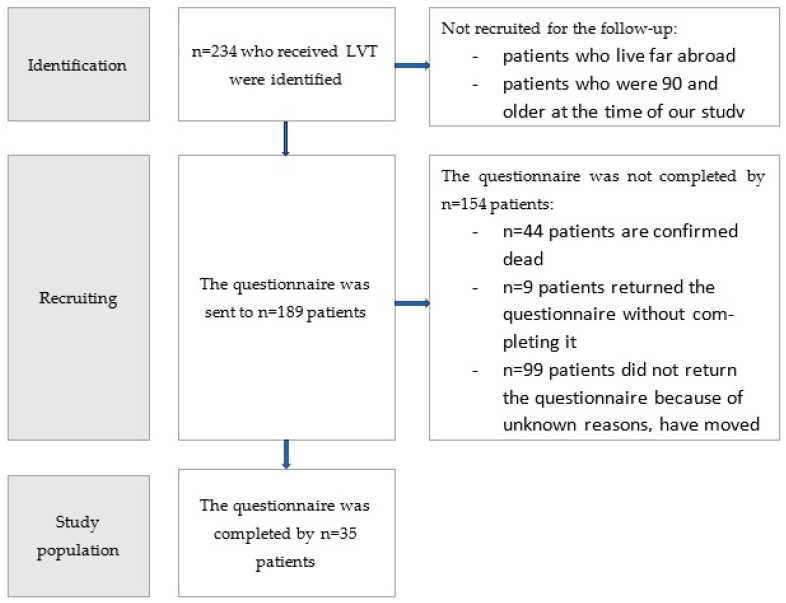
Flowchart of the study process.

The exclusion criteria encompassed an unbreachable language barrier or the patient’s refusal to participate in the study. Ethical approval was obtained from the Ethics Committee of the LMU Munich prior to the commencement of the study (Project: 23-0147) (Figure 1).

Patients meeting inclusion criteria were identified through our electronic hospital information system and the manual revision of old paper records. They were contacted via mail or e-mail if available.

**Figure 2:**
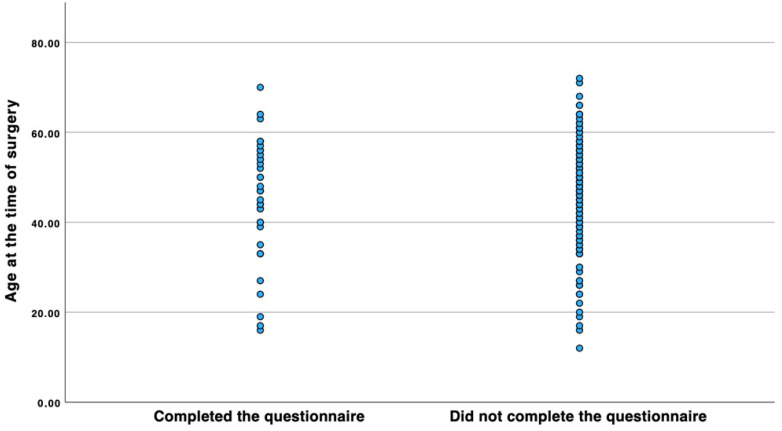
Scatter plot comparing the age distribution at the time of surgery between the cohort that completed the questionnaire and the cohort that did not. A Mann–Whitney U-test shows that the mean rank is 92.64 for the group that completed the questionnaire and 95.54 for the cohort that did not complete the questionnaire. The test statistic yielded a U-value of 2777.50 and a Z-value of 291.94, resulting in a *p*-value of 0.777.

**Figure 3:**
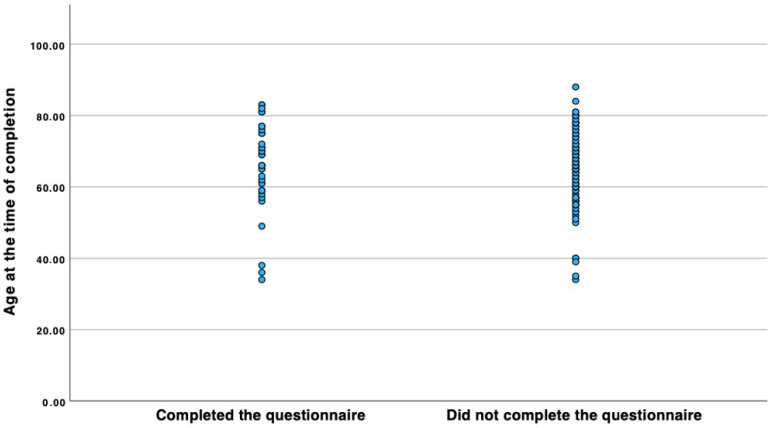
Scatter plot comparing the age distribution at the time of completing the questionnaire (we have set 1 January 2024 as the cut-off date for the cohort that did not complete the questionnaire) between the cohort that completed the questionnaire and the cohort that did not. A Mann–Whitney U-test shows that the mean rank is 77.49 for the group that completed the questionnaire and 71.57 for the cohort that did not complete the questionnaire. The test statistic yielded a U-value of 1768.0 and a Z-value of 216.23, resulting in a *p*-value of 0.468.

Questionnaires were sent to 189 patients by mail. Due to the long latency period since the operation, the dropout rate was 80%. The total study population comprised 35 patients. Patients included in the study had undergone LVT on average 21.71 ± 4.93 (mean ± standard deviation) years ago. Of the 35 patients included in the study, 17 had lymphoedema of the upper limb, and 18 had lymphoedema of the lower limb. Six patients had primary and 29 had secondary lymphoedema. Of the patients who completed the questionnaire, 4 were male and 31 were female. All patients had undergone at least 6 months of unsuccessful conservative therapy before surgery. On average, the conservative therapy was undertaken 4.79 ± 3.12 (mean ± standard deviation) years prior to the operation. In order to examine the association between the cohort who completed the SF12 questionnaire and the other patients included in the study with regard to their lymphoedema, age at the time of surgery, age at the time of completing the questionnaire, and gender distribution within the cohort, and thus to assess whether the cohort of patients who completed the questionnaire constituted a representative sample, the Chi-squared test of independence was performed. Other factors that would have been interesting for analyzing the representativeness of the cohort can unfortunately no longer be reconstructed retrospectively. The analysis included 189 patients, of which the cohort of patients who completed the questionnaire comprised 35, and the cohort of lymphoedema patients who did not complete the questionnaire comprised 154 patients. The results with regard to the gender of the patients yielded a *p*-value of 0.87 (Table 1). As a particularly large number of lymphoedema cases used to occur as part of surgical treatment for breast cancer and the associated removal of the lymph nodes, our study cohort includes a particularly large number of female patients. It is estimated that women in Central Europe are affected by lymphoedema around 4.5 to 6.1 times more frequently than men [8]. The results regarding the entity of lymphoedema gave a *p*-value of 0.651 (Table 2). A Mann–Whitney U-test was performed to determine the differences between the age at the time of surgery and when filling out the questionnaire for the patient group that completed the questionnaire (*n* = 35) and the cohort that did not (*n* = 154) (Figure 2 and Figure 3). The results indicate that there is no significant difference between the two cohorts with regard to the two characteristics analyzed. A total of 44 patients are known to be dead at the time of this writing. Unfortunately, it is no longer possible to evaluate the cause of death of these patients, as some of them were undergoing treatment in our center exclusively for LVT. However, based on the respective ages of the patients who died, it can be inferred that they died a natural age-related death. Since the majority of patients are cancer patients, death in connection with the primary disease is quite conceivable (Table 3). Overall, the results show that the cohort of patients who completed the questionnaire did not differ significantly from the overall cohort of patients who completed the questionnaire in terms of their lymphoedema, age at the time of surgery, age at completion of the questionnaire, and gender composition. Since the Chi-squared test did not reveal a significant correlation between the occurrence of the characteristic and membership in one of the two cohorts for any of the characteristics analyzed, the sample can be regarded as representative.

### Assessment Instrument

A modified SF-12 questionnaire was used to assess HRQoL. The SF-12 is a validated instrument for the measurement of HRQoL consisting of 12 questions targeting the various aspects known to influence HRQoL.

The modified SF-12 questionnaire was specially adapted to the known symptoms of lymphedema as part of a short-term follow up study on patients with lymphoedema [25]. The questionnaire refers to common symptoms and the resulting impairments and problems of patients with chronic lymphoedema that negatively affect their HRQoL. As this specific questionnaire was already used before, we adopted the questionnaire in order to create better comparability with existing data.

The questionnaire contains 10 questions, each of which discusses the preoperative and postoperative condition. Four questions relate to the physiological conditions, more specifically to the extent to which patients suffer from the feeling of pressure and tension, changes in volume during the day, pain, and acute skin infections caused by lymph stasis. Four further questions discuss the psychological conditions and refer to known problems faced by patients with chronic lymphoedema. In each case, the restriction of everyday activities, problems in partnerships and relationships, difficulties in finding clothes, and problems with stigmatization due to lymphoedema are to be discussed. Two further questions relate to the burden of therapy. Here, it is discussed how often the patient had to wear compression stockings and how often manual lymphatic drainage was necessary. All 10 questions were asked once in relation to the preoperative condition and once in relation to the postoperative condition.

The analysis was performed for the entire patient cohort and respectively for patients with lower and upper limb lymphoedema. The demographic parameters were correlated with the data obtained from the SF-12 questionnaires and information previously collected from a data register, such as the primary disease, pre- and postoperative manual measurements of the edemas, pre- and postoperative lymph vessel scintigraphies, previous treatment, and medical history. The evaluation of the results was examined with IBM^®^ SPSS^®^ Statistics using standard statistical procedures. A significant correlation is assumed here with a *p*-value < 0.05.

**Figure 4:**
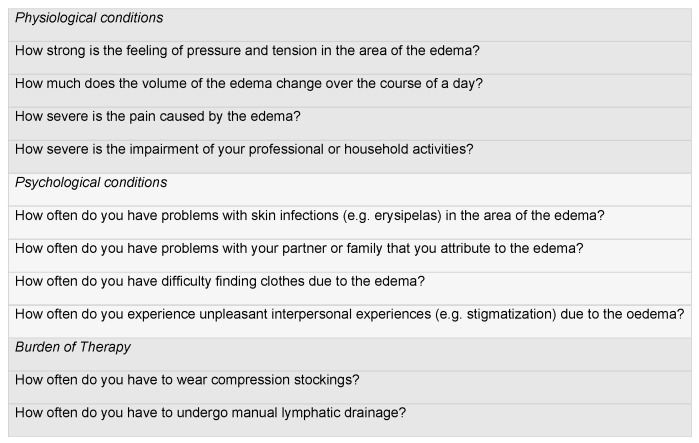
Adapted SF-12 questionnaire showing the 10 questions asked (the background color indicates different subtopics).

Each question of the adapted SF-12 questionnaire we used was given 1–5 points, with 5 points being associated with a particularly high burden and 1 point with a particularly low burden (Figure 4). The maximum number of points that could be achieved was 50, with 20 points each for the physiological and psychological conditions and 10 for the burden of therapy. A reduction in the score therefore represents an improvement in the burden on the patient and an associated improvement in HRQoL. We analyzed the whole patient cohort and patients with upper and lower limb lymphedema separately (Figure 1).

## 3. Results

### 3.1. Physiological Conditions

**Figure 5:**
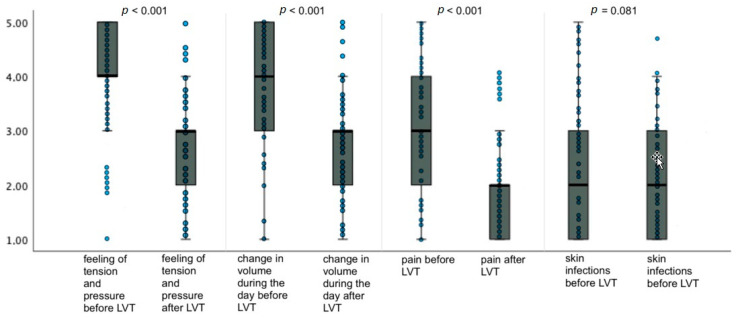
Changes in HRQoL regarding physiological conditions as asked in the adapted SF-12 questionnaire for patients with lymphoedema after LVT. Data are represented as the 25th to 75th percentiles (boxes) with the median (line in the box) and data points in the form of a scatter plot. The *X*-axis shows the respective category of the questionnaire pre- and postoperatively. The *Y*-axis shows the score given by the patients (from 1 to 5). The *p*-value was determined using the *t*-test for independent samples.

The total HRQoL value related to the physiological conditions given by the patients with upper and lower lymphedema for the condition before the operation was 12.8 ± 2.3 (mean ± standard deviation). In relation to the current point in time, meaning 21.71 ± 4.93 (mean ± standard deviation) years post operation, the patients gave a value of 9.5 ± 1.8 (mean ± standard deviation) for the physiological conditions, which indicated a highly significant improvement in HRQoL of 3.3 points (*p* < 0.001) (Figure 5).

In addition, we analyzed patients with upper and lower limb edema separately.

In the patients with upper limb lymphedema, the mean preoperative score reported by patients for physiologic conditions was 13.1 ± 3.5 (mean ± standard deviation), and the postoperative score at this time point was 9.5 ± 2.6 (mean ± standard deviation), so there was a highly significant improvement in HRQoL score of 3.6 points (*p* < 0.001).

In patients with lower limb lymphedema, the preoperative score for physiologic conditions was 12.5 ± 3.6 (mean ± standard deviation), and the postoperative score was 9.5 ± 3.0 (mean ± standard deviation). There was a highly significant improvement of 3.0 points in the HRQoL score (*p* < 0.001).

### 3.2. Psychological Conditions

**Figure 6:**
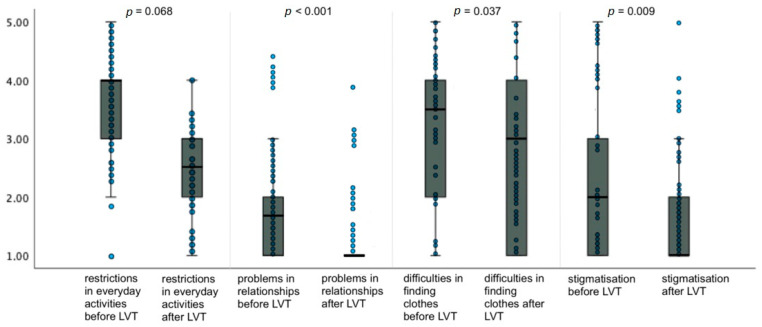
Changes in HRQoL regarding psychological conditions as asked in the adapted SF-12 questionnaire for patients with lymphoedema after LVT. Data are represented as the 25th to 75th percentiles (boxes) with the median (line in the box) and data points in the form of a scatter plot. The *X*-axis shows the respective category of the questionnaire pre- and postoperatively. The *Y*-axis shows the score given by the patients (from 1 to 5). The *p*-value was determined using the *t*-test for independent samples.

The total HRQoL value for psychological conditions reported by the patients with upper and lower lymphedema for the condition before surgery was 10.3 ± 2.0 (mean ± standard deviation). Relative to the current time point, meaning after 21.71 ± 4.93 (mean ± standard deviation) years, patients reported a score of 8.2 ± 2.1 (mean ± standard deviation) for physiological conditions, resulting in a highly significant improvement in HRQoL of 2.1 points (*p* < 0.001) (Figure 6).

In addition, we analyzed patients with upper and lower limb edema separately.

In the patients with upper limb lymphedema, the reported preoperative score for psychological conditions was 11.0 ± 3.5 (mean ± standard deviation), and the postoperative score at this time point was 8.2 ± 2.5 (mean ± standard deviation), resulting in a highly significant improvement in HRQoL of 2.8 points (*p* < 0.001).

In patients with lower limb lymphedema, the preoperative score for physiologic conditions was 9.7 ± 3.2 (mean ± standard deviation), and the current score was 8.2 ± 3.5 (mean ± standard deviation). An improvement in the HRQoL score of 1.5 points with moderate evidence (*p* = 0.025) was observed.

### 3.3. Burden of Therapy

**Figure 7:**
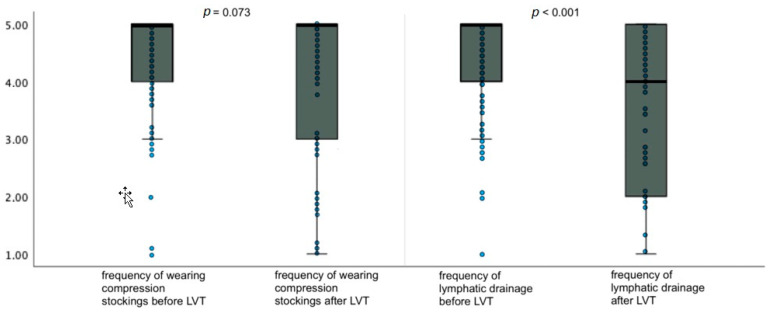
Changes in HRQoL regarding the burden of therapy as asked in the adapted SF-12 questionnaire for patients with lymphoedema after LVT. Data are represented as the 25th to 75th percentiles (boxes) with the median (line in the box) and data points in the form of a scatter plot. The *X*-axis shows the respective category of the questionnaire pre- and postoperatively. The *Y*-axis shows the score given by the patients (from 1 to 5). The *p*-value was determined using the *t*-test for independent samples.

The total HRQoL value for the burden of therapy reported by the patients with upper and lower lymphedema before surgery was 9.2 ± 1.2 (mean ± standard deviation). Relative to the current time point, meaning after 21.71 ± 4.93 (mean ± standard deviation) years, patients reported a score of 7.6 ± 1.9 (mean ± standard deviation) for physiological conditions, which indicates a highly significant improvement in HRQoL of 1.6 points (*p* < 0.001) (Figure 7).

In addition, we analyzed patients with upper and lower limb edema separately.

In the patients with upper limb lymphedema, the reported preoperative score for the burden of therapy was 9.2 ± 1.1 (mean ± standard deviation), and the postoperative score at this time point was 7.2 ± 2.4 (mean ± standard deviation). The HRQoL score improved significantly by 2.0 points (*p* = 0.002).

In patients with lower limb lymphedema, the preoperative score for physiologic conditions was 9.1 ± 1.2 (mean ± standard deviation), and the current score was 7.9 ± 2.1 (mean ± standard deviation). There was an improvement in the HRQoL score by 1.6 points with little evidence (*p* = 0.071).

## 4. Discussion

### Summary of Results and Interpretation

This study analyzes the long-term results regarding quality of life after LVT in patients with chronic lymphedema unresponsive to conservative therapy. As patients with chronic lymphoedema suffer from the symptoms and the resulting impairments, especially in everyday life, we are convinced that the HRQoL is a good tool for evaluating the results of surgical interventions. It has also already been described that the classic instruments for objective evaluation do not always correspond with the patient’s perception, but rather, PROMs should be considered more detailed [26]. Patient-centered outcomes are an increasingly popular method for assessing the results of surgical interventions. They have become an important endpoint for the assessment of the quality of healthcare and will continue to gain importance in the context of intervention decisions in the future [28,29,30]. We therefore emphasize the importance of PROMs for evaluating HRQoL in relation to treatment decisions. Especially in the case of chronic lymphoedema, the symptoms of which are known to be a major burden in everyday life, we consider an assessment of the HRQoL and the results obtained from it, which are then included in treatment decisions, to be particularly important [31].

The modified SF-12 questionnaire we used for this study is easy to use for the patient and, with its four questions each on physical and mental health and two questions on the burden of therapy, it is a good tool for evaluating and monitoring HRQoL in patients with lymphoedema both pre- and postoperatively [25].

Based on the evaluation of the modified SF-12 questionnaires for the assessment of HRQoL, a significant to highly significant improvement was found in all cases in relation to the physiological conditions, psychological conditions, and the burden of therapy. The results refer to a period of 22.71 ± 4.19 years and thus represent a long postoperative period. These results fit well with previous findings regarding postoperative HRQoL after LVT, which evaluated a shorter postoperative period. A short-term follow up study published in 2011 showed significant improvements in HRQoL shortly after LVT [25]. A limitation of our study results from the high dropout rate due to the long follow-up period and the resulting patient cohort. It is possible that after the long latency period, only patients who were particularly satisfied or particularly dissatisfied with the surgical results participated in our study. We accepted this possible selection because a long postoperative period was important to us for assessing the long-term postoperative results. A further limitation of the study is that errors in data collection are not very unlikely. Filling out the questionnaire is based on very subjective parameters and therefore also prone to bias. In addition, there is no comparison with the HRQoL of patients who have only received conservative therapy over a long period of time.

The improvement in physiological conditions in patients with upper limb lymphoedema led to an improvement of 3.6 points in the long term and 4.7 points in the short to medium term post operation [25]. In patients with lymphoedema of the lower limbs, there was an improvement of 3.0 points in the long term and 2.2 points in the short to medium term post operation [25]. Compared to the short- to medium-term results, the improvement was less positive in patients with upper limb lymphoedema but was higher in patients with lower limb lymphoedema. In the long term, the results were more similar, showing an overall significant improvement of 3.3 points. The postoperative improvement in the psychological condition of patients with upper limb lymphoedema showed an improvement of 2.8 points in the long term and 4.6 points in the short to medium term [25]. In patients with lymphoedema of the lower limbs, an improvement of 1.5 points was observed in the long term and 1.6 points in the short to medium term [25]. Compared to the short to medium term post operation, there is a less pronounced but still significant long-term improvement in patients with upper limb lymphoedema. For lower limb lymphoedema, there is an improvement in the QoL score of around 1.5 points in the long and medium term. The improvement in the burden of therapy in patients with upper limb lymphoedema resulted in an improvement of 2.0 points in the long-term and 2.6 points in the short- to medium-term postoperative period [25]. In patients with lower limb lymphoedema, a postoperative improvement of 1.2 points was found with weak evidence in the long term, and no significant improvement was found in the short to medium term [25]. The long-term improvement in the burden of therapy in patients with upper limb lymphoedema is less pronounced than in the short to medium term. Regarding patients with lower limb lymphoedema, the values showed no pronounced significance.

Overall, the long-term results in terms of improvement in quality of life are similar to the short- to medium-term results. However, it can be observed that patients with upper limb lymphoedema show better results in all categories in the medium term than in the long term. This suggests that patients with upper limb lymphoedema benefit more from surgery in the short to medium term than in the long term, and this should be considered in the treatment decision. However, the values in patients with upper limb lymphoedema are already higher before surgery than in patients with lower limb lymphoedema. These observations could be explained by the fact that lymphoedema and compression stockings on the upper limbs are more visible in everyday life, leading to more problems with stigmatization and other issues [25]. The impairments in everyday life are also more drastic for the upper extremities than for the lower extremities, at least in our Western world [32,33].

The results are very compatible with the results of objective measurement methods, such as manual volume measurement and lymphatic drainage scintigraphy. Various studies have already shown an improvement in edema after LVT [22,24,34].

A literature review from 2022 identified a total of 39 questionnaires that were used to evaluate HRQoL in patients with lymphoedema and their postoperative outcomes [35]. Nevertheless, it should be emphasized that all questionnaires evaluated in this review, including the questionnaire we used, do have their limitations [35]. This also includes the adapted SF-12 questionnaire we used within our study. Due to the heterogeneity of the tools, the question arises as to what extent the questionnaire we used for evaluation actually measures what we aimed to find out with this study. The 10 questions are well targeted at known problems and limitations in patients with lymphoedema, and we are therefore convinced that they can evaluate the HRQoL well. However, the heterogeneous range of assessment tools can make a comparison with the results of other surgical techniques more difficult. A literature review from 2021, which compared HRQoL in patients with lymphoedema after various surgical interventions, found an improvement in HRQoL for all treatment options. However, the comparison of the different surgical techniques with regard to HRQoL was difficult due to the great heterogeneity of the assessment instruments [36].

It is also important to recognize that HRQoL is also dependent on various other factors. It is significantly linked to the social determinants of health (SDHs). The SDHs are environmental factors that are related to the well-being of individuals and have an influence on the likelihood of an individual having good health. The SDHs include socioeconomic status (i.e., income and occupation), education and the associated understanding of health, as well as the physical environment (such as clean air, access to healthy food, etc.) and the social environment (the social network, etc.) [37,38]. Additionally, the HRQoL is also influenced by further factors. These include psychological factors such as self-efficacy or coping resources, but the patient’s health behavior, such as physical activity, also has an influence on HRQoL [39]. However, as all of these factors are relatively unspecific for the disease and therefore also for the success of treatment, we only asked about the disease-specific factors with the intention of improving medical care for chronic lymphoedema. In addition, certain disease-unspecific factors that influence HRQoL could no longer be collected retrospectively due to the long latency period. For holistic patient care, however, it must be recognized that HRQoL is influenced by both disease-specific factors and non-specific factors such as SDHs.

## 5. Conclusions and Implications for Further Research

The results of this retrospective study underline the effectiveness of LVT for the surgical treatment of chronic lymphoedema in terms of HRQoL. Future research is yet needed to evaluate the long-term results of LVT with a bigger patient cohort, for example as part of a meta-analysis. The results could also be compared with a control cohort in order to obtain an even more significant picture. Future research should also compare the long-term outcomes of both LVT and other surgical and conservative treatment options. In the future, it may also be important to investigate HRQoL after the surgical treatment of lymphoedema not only in relation to disease-specific factors but also non-specific factors that could contribute to treatment success. In particular, it could be helpful to develop a universal tool to assess HRQoL for patients with lymphoedema. A standardized, validated tool could improve the comparability of results with regard to HRQoL in chronic lymphedema. Prospective studies with constant and simultaneous monitoring of all patients with different therapies, in which volume increases are correlated with HRQoL, could furthermore be helpful in order to compare different therapy options. This may ultimately improve the state of research on the best treatment options in terms of objective and subjective parameters, which could help doctors to provide well-founded treatment recommendations in the future.

## Figures and Tables

**Table 1:** Cross table on gender distribution.

	Patients Who Completed the Questionnaire	Patients Who Did Not Complete the Questionnaire	Total
Male	7	29	36
Female	28	125	153
Total	35	154	189
*p*-value	*p* = 0.87		

**Table 2:** Cross table on the entity of the lymphoedema.

	Patients Who Completed the Questionnaire	Patients Who Did Not Complete the Questionnaire	Total
Primary lymphoedema	6	23	29
Secondary lymphoedema	29	131	160
Mammary carcinoma	19	61	80
Cervical carcinoma	6	32	38
Ovarian carcinoma	1	13	14
Malignant melanoma	1	6	7
M. Hodgkin	0	3	3
Other malignant tumors	0	6	6
Idiomatic genesis	0	5	5
Traumatic genesis	1	3	4
Total	35	154	189
*p*-value	*p* = 0.651		

**Table 3:** Gender, primary diagnosis, age at time of death, age at time of diagnosis, survival since diagnosis, age at time of surgery, and survival since surgery listed for the 44 patients who are known to have died.

Gender	Primary Diagnosis	Age at Time of Death	Age at Time of Diagnosis	Survival Since Diagnosis	Age at Time of Surgery	Survival Since Surgery
f	Ovarian carcinoma	56	45	11	47	9
m	Traumatic genesis	51	24	27	26	25
f	Mammary carcinoma	56	41	15	44	12
f	Mammary carcinoma	73	53	20	55	18
f	Mammary carcinoma	63	49	14	53	10
f	Ovarian carcinoma	64	43	21	47	17
f	Mammary carcinoma	58	38	20	42	16
f	Mammary carcinoma	65	46	19	49	16
f	Mammary carcinoma	62	43	19	47	15
f	Cervical carcinoma	58	44	14	47	11
f	Mammary carcinoma	75	51	24	55	20
f	Mammary carcinoma	52	39	13	43	9
f	Cervical carcinoma	51	35	16	39	12
f	Endometrial carcinoma	62	41	21	47	15
f	Cervical carcinoma	58	37	21	47	11
f	Mammary carcinoma	77	47	30	49	28
f	Mammary carcinoma	73	46	27	49	24
m	Primary lymphoedema	59	22	37	36	23
m	Ewing`s sarcoma	48	24	24	37	11
f	Primary lymphoedema	53	19	34	34	19
f	Mammary carcinoma	69	57	12	62	7
f	Mammary carcinoma	72	48	24	52	20
f	Mammary carcinoma	71	51	20	53	18
m	Malignant melanoma	82	76	6	77	5
f	Mammary carcinoma	78	69	9	74	4
f	Mammary carcinoma	69	39	30	53	16
f	Ovarian carcinoma	77	71	6	73	4
f	Mammary carcinoma	79	55	24	60	19
f	Mammary carcinoma	93	65	28	72	21
f	Mammary carcinoma	67	53	14	58	9
f	Mammary carcinoma	86	63	23	75	11
f	Malignant melanoma	79	51	28	54	25
f	Mammary carcinoma	76	61	15	64	12
f	Mammary carcinoma	71	47	24	51	20
f	Mammary carcinoma	75	63	12	69	6
f	Mammary carcinoma	65	49	16	52	13
f	Mammary carcinoma	83	64	19	73	10
f	Mammary carcinoma	68	47	21	50	18
f	Mammary carcinoma	77	60	17	65	12
m	Primary lymphoedema	78	21	57	36	42
f	Mammary carcinoma	80	62	18	71	9
f	Idiomatic genesis	84	35	49	52	32
f	Mammary carcinoma	89	53	36	56	33
f	Mammary carcinoma	71	56	15	58	13

## Data Availability

The original data presented in the study are openly available in PubMed at DOI.
